# A ‘Cut-Down-To-Stop’ intervention for smokers who find it hard to quit: a qualitative evaluation

**DOI:** 10.1186/s12889-019-6738-9

**Published:** 2019-04-15

**Authors:** J. Robinson, A. McEwen, R. Heah, S. Papadakis

**Affiliations:** 1 School of Social and Political Sciences, University of Glasgow, Florentine House, 53, Hillhead St, G12 8QS, Glasgow, UK; 2 National Centre for Smoking Cessation and Training (NCSCT), 1 Great Western Industrial Centre, Dorchester, DT1 1RD UK; 3 Department of Law, University of Liverpool, Eleanor Rathbone Building, Bedford Street South, L69 7DT, Liverpool, UK; 4 Sophia Papadakis, Division of Prevention and Rehabilitation, University of Ottawa Heart Institute, 40 Ruskin Street, Ottawa, Ontario K1Y4W7 Canada

**Keywords:** Smoking cessation, Cut Down To Stop, Qualitative evaluation, Focus groups, United Kingdom

## Abstract

**Background:**

English Stop Smoking Services primarily deliver behavioural interventions to support abrupt quit attempts. Recent evidence suggests an alternative approach could be offered to clients involving a more gradual reduction of cigarettes smoked leading to complete abstinence, known as ‘Cut Down To Stop’ (CDTS). The purpose of this study was to explore the experiences of stop smoking practitioners and service users who participated in a pilot study of a CDTS service.

**Methods:**

The CDTS intervention was pilot tested in a Stop Smoking Service in London, England. As part of the CDTS intervention clients who were still smoking 2 weeks after their quit date were offered tailored advice, medication and support to reduce their current smoking by half, with the aim to stop smoking altogether within a six-month period. A qualitative evaluation was conducted involving a focus group discussion with nine practitioners involved in the delivery of the CDTS intervention and telephone interviews with 18 CDTS service users. Thematic analysis was performed.

**Results:**

Service users and practitioners were very positive about their experience with the CDTS intervention. The intervention was found to be an effective way of keeping clients engaged with the service and was felt to increase the likelihood they might quit and/or re-engage in service for future quit attempts. Elements that contributed to the attractiveness of the CDTS intervention included: 1) the trust and empathetic relationship developed between service users, practitioners and their referring primary care provider; 2) time and flexibility for service users to engage in the quitting process at their own pace; 3) setting progressive goals and building service user confidence; 4) the opportunity to experiment with quit smoking medications; and, 5) the on-going contact with the practitioner/service.

**Conclusions:**

Service users who are not successful with quitting abruptly may benefit from a CDTS intervention. This study highlights the important role of ‘relationships’, time and ‘flexible’ service delivery models in engaging service users who are not initially successful with quitting. The findings of this study have the potential to inform decision-making regarding the value of the CDTS approach for the English Stop Smoking Service and cessation services worldwide.

## Background

While rates of smoking in the United Kingdom have dropped to approximately 15%, an estimated 7.4 million residents continue to smoke [1]. Rates of smoking remain at over 25% in some populations that are associated with social and economic disadvantage [1]. The most effective method of quitting available to smokers is a combination of medication and behavioural support [2, 3]. Most areas in England have local authority stop smoking services (SSS) that provide behavioural support and access to medication; these services are free to the public and stop smoking medications are free to those on low incomes [2, 3]. In 2016–2017, over 300,000 smokers set quit dates with local SSS [4]. Around 51% of service users self-reported successfully quitting (36.8% co-validated) 4 weeks after their quit date [4]. Approximately 27% of SSS service users failed to stop smoking within the 4 week period after their quit date, and a further 23% were categorised as lost to follow up [4].

English SSSs generally provide support over six sessions which conclude 4 weeks post quit date and emphasize abrupt quitting [3]. Clients that have not been successful in quitting may not be offered further support to continue and extend their current quit attempt, but instead are advised to set a new ‘quit date’, again focussed on abrupt cessation. This is the case for people who may have reduced their smoking substantially but not quit, and who are known to be at risk of resuming their former smoking consumption. While some SSS may extend beyond the standard four-week post-quit date service delivery period, this is the exception and not the norm [5].

Given that more than half of service users are not successful in quitting at 4-weeks there is a need to examine how best to support this group of clients with quitting. While current service provision meets the needs of some people who want to quit, it is of critical importance to consider how the needs of smokers who may fail to quit, are lost to the service and/or who resume smoking within 4 weeks can be better served. This is consistent with the National Institute for Health and Care Excellence (NICE) Guidance (2016) which recommends service providers continue to work with clients offering counselling and pharmacotherapy beyond the 4-week period [6]. Specifically, the challenge is not only to continue to attract people into English SSS, but also to sustain their engagement with services over time and support their cessation goals [7]. This is particularly important for those who have previously made a quit attempt but have either been unsuccessful or have since relapsed, and as such may be reluctant to reengage with services [8, 9].

Recent evidence suggests an alternative approach should be offered to clients of SSS known as ‘Cut Down to Stop’ (CDTS) or “Reduce to Quit” [10]. Rather than focussing on abrupt abstinence, the CDTS approach involves a reduction of cigarettes smoked over time, leading to complete abstinence. The CDTS model uses progressive goal setting to help clients unable or unwilling to quit to gain experience with the process of quitting. Evidence has shown the combination of behavioural support and a first line quit smoking medication (e.g. nicotine replacement therapy or varenicline) as part of the CDTS approach can significantly increase rates of successful quitting [10]. The purpose of this study was to explore the experiences of both stop smoking practitioners, as well as SSS service users, who participated in a pilot study of a CDTS intervention for individuals who have failed to stop smoking using the standard English SSS model. Findings of this study will provide insight regarding the role of the CDTS in meeting the cessation needs of clients unable to quit and inform future policy regarding English SSS delivery.

## Methods

### Design and setting

We conducted a qualitative evaluation of the *CDTS* intervention involving focus group discussion with nine stop smoking practitioners (‘practitioners’) and telephone interviews with 18 service users (‘clients’). The evaluation took place between May and July 2016. The study was approved by the School of Law and Social Justice Ethical Review Board at the University of Liverpool. The CDTS intervention was implemented by the Kick-It SSS (Kick It; https://www.kick-it.org.uk/.), which delivers services to three boroughs (Hammersmith & Fulham, Kensington & Chelsea, and City of Westminster) in London, UK. SSSs were delivered in smoking cessation clinics in over 100 GP practice clinics and in other community settings. Although the three boroughs are considered to be relatively affluent, there are areas of significant deprivation and the adult smoking prevalence is higher than the national rate [11].

#### Intervention

**Fig. 1:**
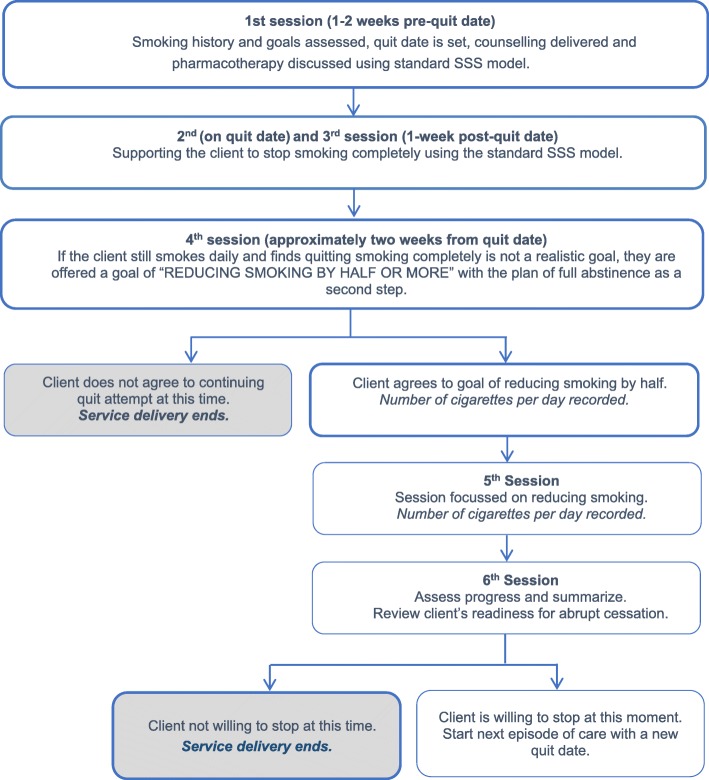
Kick It ‘Cut Down to Stop’ Intervention Protocol

CDTS intervention offered service users who are still smoking 2 weeks after their quit date further tailored support, medication and counselling to enable them to reduce their current smoking by half, with the aim of setting a new quit date, and stopping smoking altogether within a 6 month period. The CDTS intervention specifically aimed to boost service users’ motivation by refocusing their goal to ‘reduce by half’ their daily cigarette consumption in the short term, and work toward complete cessation as a second step. The intervention protocol is found in Fig. 1**.** All service users received three sessions (one pre-quit, one on the quit date, and one one-week post-quit) focussed on achieving total abstinence as per the standard English SSS [3]. During a fourth session (two-weeks post-quit date), service users who were still smoking 2 weeks following their quit date were offered the opportunity to set a new goal of smoking reduction. Service users received two additional sessions (5th and 6th session) with the stop smoking practitioner, which focussed on reducing daily cigarette consumption by half, with the aim of setting a new quit date, and stopping smoking altogether within a 6 months period. Support from the practitioner continued for an additional 2-sessions (10–12 weeks) for individuals who wished to use the CDTS approach.

During these sessions service users were provided with behavioural support and continuation of medication accompanied by tailored advice on dosage to achieve a minimum of 50% reduction. Practitioners were able to tailor support based on the service users personal readiness and set progressive goals and encouraged clients to return to the service regardless of their success with achieving these goals (See Table 1). All stop smoking practitioners were certified by the UK National Centre for Smoking Cessation and Training (www.ncsct.co.uk). First line quit smoking medications (i.e. Nicotine Replacement therapy (NRT), varenicline or bupropion) were recommended to service users and was available cost-free to clients on low incomes. Medication was accessed by a letter of recommendation from the SSS to the client’s General Practitioner. Service users who achieved reduction and were willing to stop completely afterwards, were able to use the standard SSS again (additional 6 sessions). For those who were happy with their level of reduction, a follow-up call was placed after 12 weeks.

**Table 1:** Protocol for the ‘Cut Down to Stop’ Service

Session	Description
1–3 Standard SSS Model	▪ Discuss, explain and assess nicotine dependency ▪ Explain CO-monitoring and take first CO-reading ▪ Discuss options for medication ▪ Set a quit date ▪ Discuss withdrawal symptoms and dealing with cravings ▪ Discuss main motivation to quit
4 Clients who are still smoking 2 weeks after quit date	▪ Provide advice and guidance on ‘Kick It’ CDTS intervention ▪ Assess client’s goal to reduce tobacco and establish readiness to change ▪ Advise on ultimate goal and benefits of total abstinence ▪ Provide information on support and treatment available ▪ Discuss withdrawal symptoms and dealing with cravings ▪ Assess current smoking behaviour, clarify amount smoked ▪ Look at previous attempts of reducing ▪ Explain approach and importance of thinking about moving on to quit altogether ▪ Measure CO reading ▪ Agree schedule to cut down ▪ Discuss preparation process and provide a summary
5	▪ Review progress and confirm goals and agree cut down schedule ▪ Assess current smoking behaviour, clarify amount smoked ▪ Confirm client has sufficient supply of quit smoking pharmacotherapy (NRT, varenicline, bupropion) is using enough of it, and understands how to use etc. ▪ Advice on changing routine and re-setting goal to reduce consumption further ▪ Address tactics for managing smoking frequency and pattern ▪ Measure CO reading ▪ Review readiness for abrupt cessation ▪ Develop commitment from client ▪ Discuss plans and provide a summary
6	▪ Check on progress ▪ Measure CO ▪ Review use of medication and supply with client ▪ Discuss withdrawal symptoms and dealing with cravings ▪ Discuss coping strategies for difficult situations with the client ▪ Review gradual quitting program ▪ Discuss coping strategy with regard to any high risk potential situations ▪ Discuss the transition to complete cessation, discuss setting quit date ▪ Provide a summary
7+	▪ Clients contacted 12 weeks after quit date to find out how client is doing. ▪ Clients who have not succeeded with full session and who are ready to set a quit date are offered an additional 6 session of support.

#### Procedures

Working closely with a contact at the SSS, all stop smoking practitioners (*n* = 9) involved in the delivery of the CDTS intervention were invited to take part in this research study by email. As the practitioners knew one another and were used to discussing elements of service delivery as a group, they elected for a focus group discussion, although all participants were offered the option of an individual face-to-face or telephone interviews should they prefer. The focus group took place at the time and place set aside for their team meeting each month and lasted around 1 h and 40 min. The session was facilitated by a member of the research team with experience leading focus group discussions. All stop smoking practitioners agreed to participate in the study and provided written informed consent. During the focus group session, all practitioners were asked about their roles and responsibilities, any challenges in terms of addressing smoking with clients; their experiences of delivering smoking cessation interventions and the targets they meet, and what they thought of the new CDTS intervention in terms of the overall goal of smoking cessation.

All clients involved in the CDTS service were invited to participate in the study and 34 agreed to be contacted. Eligibility criteria included: being over the age of 18; being a smoker (> 1 cigarettes per day); currently making a quit attempt with a SSS and having not managed to achieve CO-validated abstinence during weeks two and four post-quit. Service users who had not been able to quit smoking, or who had reduced smoking but not achieved full abstinence, were eligible for the CDTS intervention. Both the service users and the stop smoking practitioner must also have both agreed to change the clinical objective from abrupt cessation to CDTS. Service users could be using varenicline, bupropion, NRT, e-cigarettes or no medication for their quit attempt at the time of enrollment. A total of five call attempts were made to each client before categorizing them as lost. Among those who were successfully reached, 22 agreed to take part. Telephone interviews were conducted with 18 clients (*n* = 7 women, and *n* = 11 men). We were unsuccessful in reaching four clients who agreed to be interviewed. All client participants provided informed consent prior to the interview. Among participants, eight had substantially reduced their smoking, six participants had achieved abstinence and four were smoking at the same rate. Table 2 provides a summary of the demographic characteristics of participants. During the interviews the participants were asked to talk about their smoking history, their most recent quit attempt and also reflect on the nature and quality of the service they received. A standardized interview guide with open-ended questions was used to conduct all interviews. Interviews lasted around 30 min.

**Table 2:** Age and sex of service users (*n* = 18)

Age / Sex	Male	Female	Overall
26–35	2	1	3
36–45	0	0	
46–55	1	1	2
56–65	5	4	6
Over 65	3	1	4
Overall	11	7	18

#### Analysis

The focus group proceedings and the majority of the interviews were audio recorded and transcribed. Two service users did not want to have their telephone interviews audio-recorded and the researcher made detailed notes during the interview, which were later incorporated into the analysis. Focus group and client interview transcripts were entered into NVivo [12]. Codes were identified from reading and re-reading transcripts, and these were refined and revised during the analysis. Node reports were produced and analyzed through: detailed reading; content-sub-coding; text extraction; grouping and re-grouping. Illustrative quotations were extracted and, in a next step, re-checked against analysis for the final step in data reduction [12, 13]. All transcribed data and notes were analysed and coded thematically. The initial thematic analysis report was produced for discussion and validation by the research team. This led to re-categorization of some data and development of additional content. Project team members also identified the most salient quotes from the thematic analysis.

## Results

By and large CDTS service users reported high rates of daily cigarette consumption and many had smoked for an extended period of time. Several service users described an initial hesitation about engaging with their service and their ability to quit. Both stop smoking practitioners and all service users were overwhelmingly positive about their experience with the CDTS intervention, regardless of whether or not the client had managed to quit by the end of the intervention. Clients reported that they had been supported to achieve as much as they possibly could at that time and those clients who were unsuccessful with quitting were positive about contacting the service for future quit attempts. Despite initial concerns amongst stop smoking practitioners that offering an intervention involving cutting down may reduce client’s motivation and ultimate success with achieving full cessation, they felt that they were able to work with clients who would otherwise not have returned to the service and had assisted more clients to reduce or quit.

Two dimensions, time and relationships, were intertwined throughout the feedback received from both practitioners and all service users. Client participants reported that the CDTS intervention enabled them the time and support they needed to reduce and/or quit, and practitioners identified that the CDTS model enabled them to work with service users and give them the time they needed to change their smoking. We present the views and experiences of the clients and practitioners as seven overlapping and intersecting themes, summarized in Table 3.

**Table 3:** Summary of key themes

Theme	Relationships	Time
1. Trust and extending existing relationships	X	
2. Personal relationships and empathy between clients and practitioners	X	X
3. Time for clients to engage at their own pace		X
4. Setting progressive goals and building client confidence	X	X
5. Time to experiment with quit smoking medication	X	X
6. Flexibility and freedom to provide a client centered service	X	X
7. Maintaining relationships & keeping clients engaged in the quitting process	X	X

### Theme 1: trust and extending existing relationships

Service users identified that existing relationships with the general practitioners who had referred them to the SSS underpinned the positive relationship that was then established between the SSS practitioners and service users. Relationships of trust between primary care staff and their patients appeared to greatly influence the willingness of patients to try to quit and to be referred to the SSS.

*‘…it’s because of my illness, my doctor told me I must give up smoking because I might end up with heart problems… he recommended me to go the same day… and I made an appointment with give up smoking.’ (Client #15, male, 56–65 years, reduced).*

*‘My GP suggested it, and I’ve got a lovely GP, he suggested it and I was so ill at the time with this chest infection, I would have tried anything.’ (Client #9, female, 56–65 years, quit).*

The ‘Kick-It’ practitioners and service managers described how they had invested time motivating GPs, nurses and practice managers to refer clients who smoked. Ease of access and familiarity of venue was also mentioned by service users and something that encouraged them to contact the service.

### Theme 2: personal relationships and empathy

Service users identified both the personal relationship that was established with their stop smoking practitioner had been important part of helping them persevere with their quit attempts over time. Several service users specifically identified the empathetic, non-judgemental advice and support received from the SSS practitioner as being key to effectively addressing both the initial hesitation and difficulty clients experienced as part of their quitting process. These positive relationships were identified as being key to service users remaining engaged in the quitting process.

*‘I do feel it is very, very important* [relationship with practitioner] *actually, because I just can’t give up cigarettes and I want to give them up and so I need a lot of careful input. I must admit, I have received that…. but not in a bullying way, you know? ‘Oh you must give up cigarettes! Oh they are bad!’… none of that. It has been very carefully put to me how to cut down and eventually give up. Just in a very slow paced approach which I have found very, very helpful’. (Client #12, male, 56–65 years, reduced).*

*‘The smoking cessation practitioner had good empathy.’ (Client #12, male, 56–65 years, reduced).*

*‘I was quite apprehensive about the whole thing. I just kind of went there to find out a little bit more information. But when I met with, the first practitioner I saw she was extremely positive about the whole thing and she said, ‘You know what? Just give it a go… you are not going to lose anything if it doesn’t work the first time’. So she… sat me down, we made a plan… it just kind of went from there. It was building that relationship… I would make sure that I would see them once every week and just kind of catch up on how things are going, what stresses have happened during the week and how I was dealing with it and getting ideas from them how to deal with it….’. (Client #17, female, 26–35 years, quit).*

*“I’d had two very bad chest infections, I had COPD and plus these two chest infections (sighs) I couldn’t breathe basically! So I had to get a bit of help and the lady there, she was a delight, she was so kind, she was so empathic.”* (*Client #8, female, 65+ years, active smoking).*

While some service users communicated they were comfortable seeing different stop smoking practitioners over time, others identified the importance of seeing the same stop smoking practitioner each week, noting the importance of being able to establish a trusting relationship with a single practitioner.

### Theme 3: time and flexibility for clients to engage at their own pace

Participants noted an appreciation for the additional time that the extended CDTS service gave them to make their quit attempt.

*‘I met with ‘Kick It’ service in the beginning but it took a while to actually stick!’ (Client #17, female, 26–35 years, quit).*

For example, one service user explained that he had periods of success and relapse over the 6 months of *CDTS* program and is currently smoking one cigarette a day and working with the practitioner who has been supportive throughout.

*‘…very helpful and understanding, which means we set targets and which obviously after two weeks I come back and my targets are not met… well my consultant is very understanding and very helpful and every time has tried to give support for me just to go from one to zero’. (Client #18, male, 65+ years, reduced).*

Practitioners communicated their awareness that many of their clients were established smokers and would find it hard to quit, describing the magnitude of physical and mental effort required from some smokers in order to cut back on their smoking.

*‘You have to remember a lot of them are coming from the top of the mountain and they’re looking down, ‘Whoa, it’s so far, I’m not going to be able to get it’. (Practitioner #03).*

### Theme 4: setting progressive goals and building client confidence

Client’s described setting progressive reduction goals over time to be very helpful to their ultimate ability to reduce or quit smoking, and the additional time enabled them to notice the difference:

*‘It’s not feasible really to expect someone who’s smoking fifty a day to suddenly cut them out… So she didn’t suggest straightway cut out cigarettes completely… the idea is for me to stick to smoking ten cigarettes [a day]… and the next plan of that is to cut down to five’. (Client #12, female, 56–65 years, reduced).*

‘*It’s just really health-wise really … because I did manage to get down to eight or nine a day and over a week, at the end of the first week, I did, I don’t know if it’s psychological but I did notice a difference’*. (Client #*8, female, 65+ years, active smoking).*

One participant described quitting as ‘moving into new territory’, communicating that even small successes were valued achievements. Despite her current lack of success in quitting as part of the CDTS, she indicated that she would consider seeking help from the service to reduce her smoking in the future.

*‘I didn’t quit altogether, I just reduced, but it was like I was going in to new territory, you know? …on the quit date I managed to go six hours and that was a record for me you know? Six waking hours, I’m not talking about sleeping hours. I mean six waking hours and then I couldn’t stand it any more… I’d consider doing the reduction thing again... trying reducing you know.’ (Client #13, female, 56–65 years, reduced).*

Others needed time to build up their confidence in their ability to achieve reduction targets and put a plan in place that worked for them. Practitioners identified it was helpful to know that if the first attempt failed that they would be able to continue to work with the client.

*‘ Because they get that confidence and the practice [with quitting], and the medication as well, they start gaining that trust in, in the whole concept and they have that relationship with us by then’. (Practitioner #03).*

*‘Yeah… when they’ve kind of slightly got down, kind of halfway, quarter way, and they’re thinking, OK actually you know it’s not as hard as I thought. It’s not, OK maybe I can jump to quit. … But it maybe just takes longer, it’s not in the six weeks, it’s twelve weeks, it’s eighteen weeks’. (Practitioner #02).*

### Theme 5: time experiment with quit smoking medications

The additional time meant that practitioners were able to offer different nicotine replacement products to service users, who could discuss what worked and what didn’t work and then try something else. Again, the on-going relationship with the practitioner and the understanding they showed underpinned their willingness to keep trying products:

*‘She was giving me different products... for example when she gave me chewing gum, I would have a problem with my teeth. And then she gave me a patch… and she gave me something to put under my tongue… and like every week it was different medication… she was very nice and she was very kind, and she was explaining everything to me.’ (Client #15, male 56–65 years, reduced).*

*‘I was offered the various products. So I was pleased about that.’ (Client #12, male, 56–65 years, reduced).*

Practitioners were aware that many service users had memories of past products that they felt had not worked, or had been told by family and friends to avoid. Practitioners knew that they needed to address any myths and concerns about products service users had tried in the past and also spend time to offer them alternative products if they did not work for the service user. Practitioners spent time talking to service users and this enabled them to establish trust and run through the different options available.

*‘…some of them heard bad opinions about them, so they say, oh yeah, these don’t work … my friends told me they don’t work. But then when it actually comes to the programme and they start using them, start practising with them, they actually see that some of them are not that bad and they actually help, so… it makes it more likely for them to come back.’ (Practitioner #04).*

### *Theme 6:* flexibility and freedom to provide a client centred service

An important element for practitioners was flexibility and the freedom to engage with service users in different ways. Practitioners appreciated the chance to work with service users over time and not simply terminate their contact with service users. The opportunity to quit over a longer period of time added to their sense of providing a client-centred service commenting about how they felt more comfortable being able to continue the relationship without having to set a quit date immediately.

*‘…we would then have that discussion with the client and say, look, you know, with this programme unfortunately we need to terminate it… What we added just to allow people to stay with us, choosing to see us without setting up quit date straight away.’ (Practitioner #02).*

*‘(quitting smoking)… would be my ultimate goal with anybody. But you know having the, having that flexibility and being able to say to somebody and you know, you know well done, you know, you’ve got this far, you’ve done that much, like that was fantastic…’ (Practitioner #02).*

### Theme 7: maintaining relationships and keeping clients engaged in the quitting process

The stop smoking practitioners at ‘Kick It’ were aware of the importance of keeping people engaged with the service and described how that they spent time ensuring that service users knew that even if they missed an appointment, or stopped coming for some time, they would be welcomed back. This was an explicit part of their conversations with service users who were aware that they need not to worry if they decided to take a break from the service:

*‘But of course the biggest test I guess is their attendance… I will keep chasing them you know and they say I can’t, or I’m not ready, I say OK, then take a break but when you’re ready come back, you know rather than feeling, oh I didn’t go to that appointment, now I can’t go back to see* [name] … I tell them just keep coming… rather than them thinking there’s going to be an issue or something?’ (Practioner #7).

Practitioners reported that following their experience in delivering the CDTS intervention they were now confident that they could use the time effectively to help service users reduce as a precursor to quitting, and by keeping them in the service, this would be more likely to happen.

Being able to keep going was also popular with the service users we spoke to. Both participants and practitioners did not feel the CDTS lead to poorer outcomes and rather allowed them to work with a group of clients that might otherwise have reengaged with the service.

*‘I’d stopped any attempts at quitting… and I went back to meet her* [name of practitioner] *again and that’s when we started on the Champix… the minute I started taking Champix, you just kind of are put off by smoking… you end up putting them out and avoiding them and it makes you feel sick, so I ended up quitting I think before my quit date.’ (Client #17, female, 26–35 years, quit).*

*‘I’ve got the suspicion that our quit rate won’t be compromised as much because we will be bringing in people that normally wouldn’t get engaged with the service, and by doing that, we actually expose them in the first place.’ (Practitioner #02).*

*‘Yeah. And then they come back to quit eventually*.’ (Practitioner #01).

## Discussion

This study was designed to examine the experiences and perceived value of the CDTS intervention from the perspective of both service users and stop smoking practitioners. The findings of this qualitative evaluation suggest that the introduction of an intervention that enables practitioners to continue to work with service users, who are finding it hard to quit within the current four-week post-quit timeframe used by the English SSS, represents a very positive experience for both service users and practitioners. Service users involved in the CDTS were still actively working to quit even if the short-term goal was to reduce smoking. During the period CDTS support was provided, some service users managed to quit and others reduced their smoking (often substantially) over months. Those who were still smoking were positive about the likelihood that they would use the service for future quit attempts. This suggests that such an extended intervention is an effective way of keeping people engaged with the SSS and increase the likelihood that they might re-engage in the smoking service for future quit attempts.

The two overarching themes, which emerged from our research, were the important role of *time* and *relationships* in enhancing client engagement and success with quitting. Clients highlighted that the relationship they formed with a particular stop smoking practitioner was key to their success. Likewise, the CDTS service offered more time for clients to prepare and set progressive goals to reduce their smoking. Over the increased timeframe, practitioners were able to work with clients to try different quit smoking medications to find the support that worked for them, and at the same time addressing any concerns, experiment. The CDTS model was felt to be helpful in building service user confidence and avoiding the stigma and potential alienation of a ‘failed’ quit attempt [6].

Others have also reported promising findings in terms of the use of gradual cessation (CDTS) in research settings. A recent Cochrane review identified 24 studies which tested interventions to help tobacco users cut down the amount smoked with or without pharmacotherapy and reported a positive effect on reduction in cigarettes smoked per day as well as the likelihood of ultimately quitting smoking [10]. Use of NRT versus placebo also significantly increased the likelihood of ultimately quitting smoking and one trial reported on the use of bupropion and varenicline to assist with smoking reduction [10].

### Implications for practice

The empathetic and supportive approach used by stop smoking practitioners during CDTS intervention was reported as key to keeping service users engaged and working towards quitting. This is consistent with other published studies, which have looked at the important factors related to engaging tobacco users in quitting. Past research into the reasons why some people do not stop smoking suggests that impulsiveness; social-economic environment and personal crises as well as personal smoking history are critical factors [14–17]. A systematic review from the Cochrane collaboration identified that while the evidence was limited, three factors are associated with successful recruitment and engagement with smokers to assist them to quit: 1) *personal, tailored interventions; 2) recruitment methods that are proactive in nature; and 3) more intensive recruitment strategies (*i.e.*, those strategies that require increased contact with potential participants)* [18]. The nature of the delivery of motivational messages as part of supporting smokers to quit is known to influence the effectiveness of the therapeutic relationship between practitioner and client and boosting motivation is an evidence-based behaviour change technique [19, 20].

While the content of the intervention and getting the right advice are essential factors, both *when* and the *way* in which the advice is delivered appears to be important. So, the timing, tone and manner in which the intervention is delivered can make the difference between successful and unsuccessful engagement of a smoker with a smoking cessation service [21–23]. These findings communicate the important element of the personal and the social aspects of quit smoking support, and highlight the importance of relationships between stop smoking practitioners and potential quitters in the recruitment and retention of people to services. The CDTS intervention pilot tested in this study addresses the need for some service users to have a specialist service that is person-centred and works with individuals using a flexible and responsive plan that is focussed on building relationships with clients that are enduring over time.

Continuity of service, in particular the ability to work with the same practitioner, is valued by clients and can underpin successful outcomes for some clients. While continuity of a therapeutic relationship over time is important to some people, and may be compromised if they are unable to see the same practitioner, for others it is more about continuity in terms of the approach to service delivery, which was characterized as positive and enabling.

In order to engage a larger group of individuals, the criteria for accessing CDTS could be adjusted to include those who start smoking again with 4 weeks of their quit attempt. Concerns raised by practitioners in terms of viewing the CDTS as a second step for clients who fail versus a stand-alone service should be further examined, and a review of the medication that clients and advisors feel is efficacious in terms of supporting a more gradual sustained quit attempt. Some practitioners communicated a conern that offering clients the CDTS may adversely affect their performance outcomes which are specific to 4-week quit rates. It would be important for service planning to include a method for ensuring performance indicators are aligned with new approaches to service delivery in order not to create disincentives.

### Study limitations

Our study findings should be interpreted in light of its limitations. This qualitative study is limited to providing insight about the perceptions and reported experiences of nine practitioners and 18 client informants from one SSS in England, and these findings are not generalizable. Specifically, not all clients involved in the CDTS service agreed to participate in the interview process and as such study participants could be those respondents with a more positive experience using the service. Likewise a larger evaluation of the CDTS service examining quantitative outcomes such as reduction in cigarettes smoked, cessation rates, rates of client re-engagement may add to the findings of this qualitative study.

## Conclusions

Service users and practitioners involved in the pilot testing of the CDTS were positive about their experience and felt it increased the odds that the clients would cut down or quit, as well as, reengage with the SSS for future quit attempts. The additional time, flexibility provided to clients, empathetic relationships and progressive goals setting offered to CDTS services users were important factors to the service’s success and client satisfaction. Smokers who are not successful with quitting may benefit from the option of using a CDTS intervention. The findings of this pilot study have the potential to inform decision-making regarding the value of the CDTS approach for the English SSS and cessation services worldwide.

## Abbreviations

CDTS: Cut Down To Stop
NHS: National Health Services
NICE: The National Institute for Health and Care Excellence
SSS: Stop Smoking Services
